# Discriminative detection of laser-accelerated multi-MeV carbon ions utilizing solid state nuclear track detectors

**DOI:** 10.1038/s41598-021-92300-1

**Published:** 2021-08-11

**Authors:** Takamasa Hihara, Masato Kanasaki, Takafumi Asai, Tamon Kusumoto, Satoshi Kodaira, Hiromitsu Kiriyama, Keiji Oda, Tomoya Yamauchi, Wei-Yen Woon, Yasuhiro Kuramitsu, Yuji Fukuda

**Affiliations:** 1 Graduate School of Engineering, Osaka University, Suita, Osaka 565-0871 Japan; 2 Graduate School of Maritime Sciences, Kobe University, Kobe, Hyogo 658-0022 Japan; 3 Kansai Photon Science Institute (KPSI), National Institutes for Quantum and Radiological Science and Technology (QST), Kizugawa, Kyoto 619-0215 Japan; 4 National Institute of Radiological Sciences (NIRS), National Institutes for Quantum and Radiological Science and Technology (QST), Inage, Chiba 263-8555 Japan; 5 Department of Physics, National Central University, No. 300, Jhongda Rd., Jhongli, Taoyuan 320 Taiwan

**Keywords:** Characterization and analytical techniques, Plasma-based accelerators, Characterization and analytical techniques, Laser-produced plasmas, Graphene, High-field lasers, Ultrafast lasers

## Abstract

A new diagnosis method for the discriminative detection of laser-accelerated multi-MeV carbon ions from background oxygen ions utilizing solid-state nuclear track detectors (SSNTDs) is proposed. The idea is to combine two kinds of SSNTDs having different track registration sensitivities: Bisphenol A polycarbonate detects carbon and the heavier ions, and polyethylene terephthalate detects oxygen and the heavier ions. The method is calibrated with mono-energetic carbon and oxygen ion beams from the heavy ion accelerator. Based on the calibration data, the method is applied to identify carbon ions accelerated from multilayered graphene targets irradiated by a high-power laser, where the generation of high-energy high-purity carbon ions is expected. It is found that 93 ± 1% of the accelerated heavy ions with energies larger than 14 MeV are carbons. The results thus obtained support that carbon-rich heavy ion acceleration is achieved.

## Introduction

Laser-driven ion acceleration has been one of the most active areas of research^1, 2^. This is because the resultant ion beams have unique properties, such as an ultrashort duration, a high brilliance, and a low emittance. Therefore, applicability of laser-accelerated ions is actively discussed. For example, because of the larger linear energy transfer (LET) coefficient of carbon ions than that of protons, carbon ions are attractive for applications such as ion fast ignition^3^ and heavy ion therapy^4^. Thus far, laser-driven multi-MeV carbon ions have been generated using carbon-based targets such as diamond-like carbon (DLC)^5^, double-layer targets composed of carbon nanotube foam (CNF) and DLC^6^, ultrathin (10–100 nm) carbon foils^7^, and large-area suspended graphene (LSG)^8,9^. In all these experimental works, Thomson parabola (TP)-type ion energy analyzers^10–12^ have been utilized to characterize the accelerated ions, where ions pass through electric and magnetic fields and are differentiated by their mass-to-charge ratios and the energies. Here, it should be noted that in laser-driven ion acceleration using carbon-based targets, not only carbon ions, but also protons and oxygen ions from surface contaminants are also accelerated, except in the case of positive surface cleaning. The drawback of the TP method is that fully stripped ions of C^6^^+^ and O^8+^, which have the same charge-to-mass ratio of 1/2, cannot be detected separately because ions with the same charge-to-mass ratio trace the same parabola in the TP detector plane. Together with the development of target surface cleaning technique^13–15^, which remove the surface contaminants, measuring the purity of carbon ions in a laser-accelerated ion beam is crucial for developing a method to create an impurity-free multi-MeV carbon ion source. In addition, a precise quantitative characterization of ion species in the energy domain allows us to understand the physics of laser-driven ion acceleration process. Therefore, a method to discriminate carbon ions from background oxygen ions is an important issue.

Here, we propose a new diagnosis method for the discriminative detection of multi-MeV carbon ions from oxygen ions utilizing solid-state nuclear track detectors (SSNTDs). The SSNTDs have the great advantage of being able to detect only ions because they are insensitive to X-rays and energetic electrons^16^. SSNTDs such as CR-39 have been used for a long time as the reliable integral-type detectors in laser-driven ion acceleration experiments^17–21^. The most sensitive CR-39 can record protons as etchable tracks with energies less than 20 MeV and the heavier ions^22^, and other SSNTDs can detect heavy ions depending on their track registration sensitivities^23^. Since the sensitivity of the SSNTD depends on its material and manufacturing process^24, 25^, it is possible to selectively detect heavy ions using different kinds of SSNTDs^23^. For example, bisphenol A polycarbonate (PC) can detect carbon ions around the Bragg peak energy, but cannot detect alpha particles, while polyethylene terephthalate (PET) has no response to carbon ions^23, 26^. Therefore, identification of carbon ions from oxygen ions can be possible with the combination of PC and PET. Similar method using polyimide was applied to detect laser-accelerated Al ions, where polyimide, which can detect Si and the heavier ions, identified Al ions in distinction to other ions from surface contaminants produced from laser-irradiated thin Al foil^27^.

In this paper, we report on the results of the new diagnosis method utilizing the combination of PC and PET for discriminative detection of laser-accelerated multi-MeV carbon ions from background oxygen ions.

## Results and discussion

### Calibration with heavy ion beams

**Figure 1:**
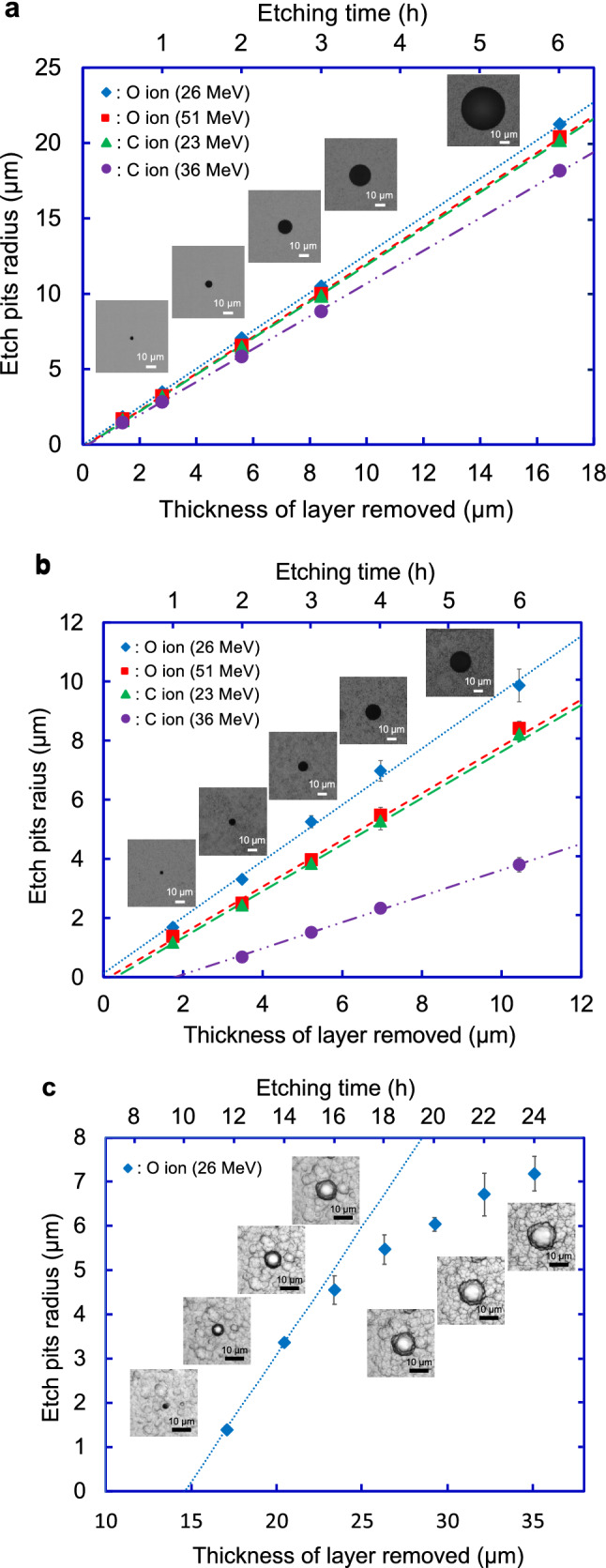
Etch pit growth curves of carbon and/or oxygen ions in (**a**) CR-39, (**b**) PC, and (**c**) PET. The inserted microscope images in (**a**)–(**c**) show etch pits of the 26 MeV oxygen ion registered on the front surface of each SSNTD at each etching time. The error bars represent the standard deviations of the etch pit radius. Some error bars are behind the markers.

Figure 1a shows the etch pit growth curves of carbon and oxygen ions, and the optical microscopic images of etch pit of oxygen ion obtained using CR-39. All the curves in Fig. 1a cross the origin, which confirms that the etch pits were created from the surface of CR-39. The radii of the etch pits linearly increase as a function of the thickness of the layer removed. Focusing on the growth curve of the 23 MeV carbon ions, the slope of it is almost equivalent to that of the 51 MeV oxygen ions. This result shows that it is not possible to distinguish carbon ions from oxygen ions from the radii of etch pits registered on CR-39 unless the ion incident energies are known^28,29^.

For PC, the etch pits of both carbon and oxygen ions are observed. Figure 1b shows the etch pit growth curves of carbon and oxygen ions, and the microscopic images of etch pit of oxygen ion obtained using PC. All the growth curves, except in the case of the 36 MeV carbon ions, cross the origin point. The result shows that the etch pits from the 36 MeV carbon ions are generated not from the surface but from the inside of the PC: From the point where the growth curve intersect the x-axis, i.e., when the 36 MeV carbon ions penetrate through 1.9 µm depth inside of the PC, the etch pits from the carbon ions become visible with the optical microscope. Therefore, from the range-energy relationship, the energy where the etch pits from carbon ions start to grow is calculated to be 35 MeV by using the SRIM code^30^. Since the slope of the 23 MeV carbon ions is almost equivalent to that of the 51 MeV oxygen ions, it is not possible to distinguish carbon ions from oxygen ions from the radii of etch pits registered on PC unless the ion incident energies are known.

For PET, the etch pit of carbon ions is not observed, even though the thickness of the layer removed exceeded the ranges of carbon ions. On the other hand, the etch pits from the 26 MeV oxygen ions are observed after 11.75 h of long etching. Figure 1c shows the etch pit growth curve of the oxygen ions and the optical microscopic images of the etch pit from the oxygen ion obtained using PET. Etch pits of the 26 MeV oxygen ions start to grow from 14.6 µm depth inside of the PET and etch pit radius increases linearly with increasing thickness of the layered removed, i.e., etching time. Then, the increasing trend of the radius is slowing down above the thickness of the layer removed of about 23 µm, meaning the start of spherical phase of etch pit evolution^31^. Since the etch pits from the 26 MeV oxygen ions become visible after the oxygen ions penetrate through 14.6 µm depth inside of the PET, from the range-energy relationship, the energy where etch pits from oxygen ions start to grow is calculated to be 6 MeV by using the SRIM code^30^.

We have confirmed that PC can detect both carbon and oxygen ions, while PET can detect only oxygen ions. Therefore, it is concluded that if PC and PET detectors are used together, it is possible to discriminatively detect carbon ions from oxygen ions in a mixed beam of those ions.

### Application to laser-driven ion acceleration experiments

**Figure 2:**
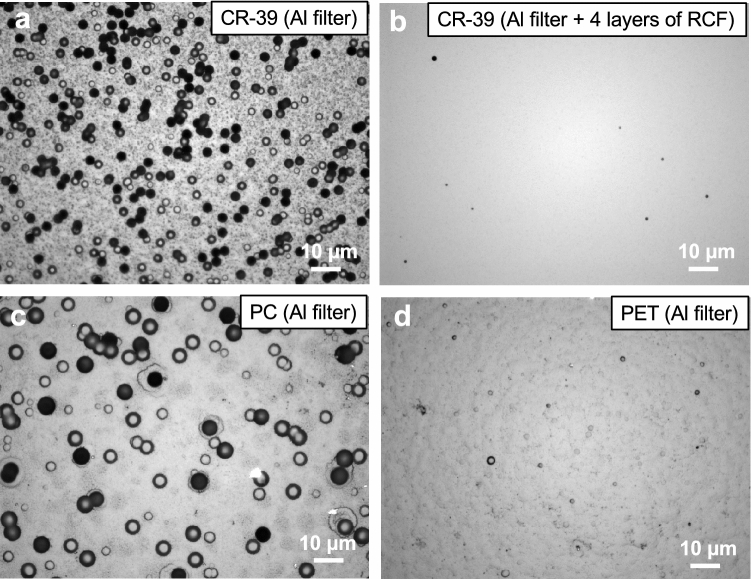
Microscope images of etch pits registered on the front surface of the first layer of (**a**), (**b**) CR-39, (**c**) PC, and (**d**) PET. (**a**) The smaller (less than 1 μm in radius) and the larger (around 2 μm in radius) etch pits correspond to protons and carbon/oxygen ions, respectively. Note that the smaller etch pits were overlapped each other. (**c**), (**d**) Surface of each SSNTD is rough because of chemical damage during etching.

Figure 2a–d show the optical microscope images of etch pits on SSNTDs. In Fig. 2a, two types of etch pits, i.e., smaller (less than 1 mm in radius) and larger (around 2 mm in radius) etch pits, are observed on the front surface of the first layer of CR-39 covered with a 12-µm thick Al filter, where the smaller and the larger etch pits correspond to protons and heavy ions, respectively. It was reported that the etch pit radii on CR-39 (TD-1) after a 30-min etching were less than 1 mm for multi-MeV protons^40^, while for multi-MeV carbon and oxygen ions the radii were around 2 mm (see Fig. 1a). On the other hand, judging from the etch pit size in Fig. 2b, only protons are detected on the front surface of the first layer of CR-39 covered with the Al filter and 4 layers of radiochromic films, which means that, according to the range-energy relationship^30^, protons with energies larger than 10.1 MeV were generated. No etch pit is observed on the front surface of the first layer of CR-39 covered with the Al filter and 5 layers of radiochromic films, which means that protons with energies larger than 11.5 MeV were not generated. Therefore, from the range-energy relationship^30^, the maximum energy of protons is estimated to be 10.8 ± 0.7 MeV.

Several etch pits from heavy ions, which pass through the 12-µm thick Al filter, are observed on the front surface of the first layer of both PC (Fig. 2c) and PET (Fig. 2d), which means that carbon and oxygen ions with energies larger than 14 and 20 MeV, respectively, were generated. Here, we assume that the carbon ions from graphene targets and the oxygen ions from surface contaminants are the major components of laser-accelerated heavy ions. This hypothesis is correct because we confirmed from a simultaneous measurement using a TP that the amounts of heavy ions other than carbon and oxygen were negligible. Since no etch pit is observed on the front surface of the second layer of both PC and PET, which means that carbon and oxygen ions with energies larger than 100 and 155 MeV, respectively, were not accelerated, the maximum possible energies of carbon and oxygen ions are estimated to be 57 ± 43 and 87.5 ± 67.5 MeV, respectively.

It is clearly understood that the number of etch pits on PC is much larger than that on PET, which indicates that carbon ions were the main product. The number of etch pits are counted as 1.34 × 10^7^ and 9.50 × 10^6^ for PC and PET, respectively. Since the number of etch pits on PC includes both of carbon and oxygen ions and that on PET includes oxygen ions only, it can be said that 1.24 × 10^7^ of the etch pits on PC belong to carbon ions. Therefore, the number ratio of the accelerated carbon and oxygen ions are evaluated to be 93 ± 1% and 7 ± 1%, respectively, where the margin of error is connected to the uncertainty of the etch pit count processes using the HspFit software^37^.

## Conclusions

We have demonstrated a new diagnostic method for the discriminative detection of multi-MeV carbon ions from oxygen ions with the combination of PC and PET, which have the different track registration sensitivities. The method was applied to a laser ion acceleration experiment using multilayered graphene targets. We found that 93 ± 1% of accelerated heavy ions was due to carbon ions with energies greater than 14 MeV, demonstrating that carbon-rich heavy ion acceleration was achieved. Since the method we developed in the present study is the one and only method which can distinguish between the fully stripped multi-MeV ions of C^6+^ and O^8+^*, i*n the future attempt to further increase the purity of the carbon ions in combination with a proper target cleaning technique, the method will an indispensable means for evaluating the purity of carbon ions.

## Materials and methods

### Calibration with heavy ion beams

The calibration for the new diagnosis method utilizing solid state nuclear track detectors (SSNTDs) was conducted at the medium energy irradiation room of the Heavy Ion Medical Accelerator in Chiba (HIMAC) in QST-NIRS, Japan. When an energetic ion enters the SSNTD, the ion transfers kinetic energy to the material and loses energy. If the deposit energy to the SSNTD is larger than the threshold, which depends on characteristics of the SSNTD material, the damaged region, which is called the ion track, is formed with a size of a few nanometer in the radial direction. The ion track region is preferentially etched by chemical etching compared to the pristine region, and then an etch pit with a size of a few microns is created, which can be recognized with an optical microscope. Each etch pit on an SSNTD shows unique growth behavior that depends on the energy and nuclide along the ion track, allowing us to predict the ion species and the incident energy by using a multistep etching technique^32–34^.

In the present study, bisphenol A polycarbonate (PC) (CT303050, Goodfellow) and polyethylene terephthalate (PET) (ES303010, Goodfellow) with a nominal thickness of 1.0 mm were used for the discriminative detection. In comparison, the most sensitive poly allyl diglycol carbonate, HARZLAS TD-1 (Fukuvi Chemical Industry), one of the most popular CR-39 detectors^35,36^, with a nominal thickness of 0.9 mm, was used as a reference. The detectors were exposed to carbon and oxygen ions with the energies below 6 MeV/u. The incident energies of ions were set to 23 and 36 MeV for carbon ions, and 26 and 51 MeV for oxygen ions. To minimize overlapping of each etch pit, the fluence was controlled at 10^4^ ions/cm^2^. After irradiation, CR-39, PC, and PET were chemically etched in a stirred 6 M KOH solution maintained at 70, 60, and 50 °C, respectively. A multistep etching technique was applied to reveal the etch pit growth behavior, and confirmed the incident energies from the ranges of ions^16,33,37^: The etch pits radius on CR-39 was measured at etching time of 0.5, 1, 2, 3, and 6 h. That on PC was measured at 1, 2, 3, 4, and 6 h. That on PET was measured at 11.75, 14, 16, 18, 20, 22, and 24 h. The bulk etch rates for PC and PET in our etching system were also evaluated to obtain etch pit growth curves from the changes in thickness for each etching time. The bulk etch rates of CR-39, PC, and PET obtained were 2.8, 1.7, and 1.5 µm/h, respectively. Microscope observations on the etched surface of each SSNTD were carried out using the fast automated digital imaging optical microscope with 20 × magnification (HSP-1000, Seiko Precision Inc.). The observed area of each SSNTD was 4.5 × 4.5 cm^2^. The size of the each etch pit open mouth and the number of the etch pits was analyzed by HspFit software^37^, where the uncertainty in the number of the etch pits was evaluated by a human operator who carefully examined a subset of full etch pit images by visual observation, while that in the etch pit radius is the standard deviation.

### Application to laser-driven ion acceleration experiments

Laser-driven ion acceleration experiments using LSG targets^8,9^ was conducted with the J-KAREN-P laser at QST-KPSI, Japan^38,39^. In the present experiment, 40 fs pulses with a pulse energy of 20 J were delivered to the experimental chamber. The laser pulses were focused with an *f*/1.3 off-axis parabolic mirror, leading to a peak intensity of 1 × 10^21^ W/cm^2^ in vacuum. LSG was developed as a laser target by National Central University (NCU)^8^. It is possible to stack multiple layers of LSG according to the objectives. We used 4-, 8-, 16-, or 32-layer LSG attached to a target holder with a 400 µm hole in the experiment. The number density of carbons per one graphene layer was 3.82 × 10^15^ cm^−2^.

**Figure 3:**
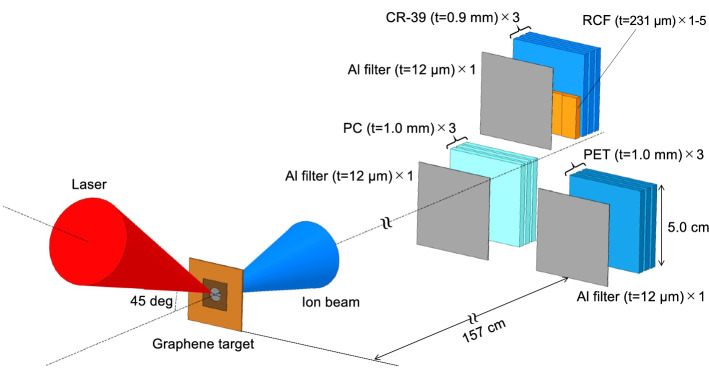
Schematic of the laser-driven ion acceleration experiment using the LSG target with J-KAREN-P laser.

In the measurements of laser-accelerated ions, three sets of stack detectors, each of which is composed of three layers of CR-39 (HARZLAS TD-1, Fukuvi Chemical Industry), PC (CT303050, Goodfellow), or PET (ES303010, Goodfellow) with the size of 5 × 5 cm^2^, were installed in the target normal direction at a distance of 157 cm from the laser focus (Fig. 3), which corresponds to a solid angle of 8 msr. From the etch pits special distribution analysis, we confirmed that the spatial inhomogeneity of the laser driven ion beams was negligibly small. The detectors were covered by a 12-µm thick Al foil to remove a large number of low energy ions. To measure the maximum energy of protons, a stepwise energy filter, which consists of five radiochromic films (RCF) (XR-RV3, GafChromic) different in size with a nominal thickness of 231 µm, was placed in front of CR-39 (Fig. 3)^40^. The minimum energies for proton, carbon and oxygen ions, which pass through the 12-µm thick Al filter, were 1, 14, and 19 MeV, respectively. After irradiation with laser-accelerated ions, CR-39 was etched for 30 min, and PC and PET were etched for 2 h. The etch pit signals thus obtained were analyzed by the same procedures described in the calibration studies.
